# Cell free DNA from respiratory pathogens is detectable in the blood plasma of Cystic Fibrosis patients

**DOI:** 10.1038/s41598-020-63970-0

**Published:** 2020-04-23

**Authors:** Sara L. Rassoulian Barrett, Elizabeth A. Holmes, Dustin R. Long, Ryan C. Shean, Gilbert E. Bautista, Sumedha Ravishankar, Vikas Peddu, Brad T. Cookson, Pradeep K. Singh, Alexander L. Greninger, Stephen J. Salipante

**Affiliations:** 1 Department of Laboratory Medicine, University of Washington, Seattle, WA USA; 2 Division of Critical Care Medicine, Department of Anesthesiology and Pain Medicine, University of Washington, Seattle, WA USA; 3 Department of Microbiology, University of Washington School of Medicine, Seattle, WA USA

**Keywords:** Genomics, Next-generation sequencing, Bacterial genetics, Clinical microbiology, Infectious-disease diagnostics, Pathogens

## Abstract

Diagnostically informative microbial cell-free DNA (cfDNA) can be detected from blood plasma during fulminant infections such as sepsis. However, the potential for DNA from airway pathogens to enter the circulation of cystic fibrosis (CF) patients during chronic infective states has not yet been evaluated. We assessed whether patient blood contained measurable quantities of cfDNA from CF respiratory microorganisms by sequencing plasma from 21 individuals with CF recruited from outpatient clinics and 12 healthy controls. To account for possible contamination with exogenous microbial nucleic acids, statistical significance of microbe-derived read counts from CF patients was determined relative to the healthy control population. In aggregate, relative abundance of microbial cfDNA was nearly an order of magnitude higher in CF patients than in healthy subjects (p = 8.0×10^−3^). 15 of 21 (71%) CF patients demonstrated cfDNA from one or more relevant organisms. In contrast, none of the healthy subjects evidenced significant microbial cfDNA for any of the organisms examined. Concordance of cfDNA with standard microbiological culture of contemporaneously collected patient sputum was variable. Our findings provide evidence that cfDNA from respiratory pathogens are present in the bloodstream of most CF patients, which could potentially be exploited for the purposes of noninvasive clinical diagnosis.

## Introduction

Cystic fibrosis (CF) is marked by chronic airway infection and progressive respiratory decline^1,2^. A key challenge in CF is accurately diagnosing the onset and persistence of lung infections. This is particularly important for the pathogen *Pseudomonas aeruginosa*, which causes pulmonary function deterioration and for which eradication regimens are effective if initiated promptly^3^. New approaches for assaying infection are most needed for children, as they are often unable to expectorate sputum^4^ and because preserving lung function is more feasible than restoring function after decline^3^. Patients receiving highly effective CFTR modulators could similarly benefit from such approaches, as that therapy reduces sputum production and can thereby confound assessments of infection status^5^.

An emerging biomarker in infectious disease diagnosis is microbial plasma cell-free DNA (cfDNA). cfDNA is highly fragmented nucleic acid released from decomposing cells into a patient’s blood, which can be identified using next-generation DNA sequencing. Although the overwhelming majority of cfDNA is derived from a patient’s own cells^6,7^, trace material from microbial pathogens can be detected from plasma during acute infection^8–15^, suggesting utility in noninvasively diagnosing pathogens. Interpretation of microbial cfDNA is complicated by contaminating bacterial DNA^8,16,17^ from molecular biology reagents and the environment^16–18^ that can cause false positives. Consequently, approaches for statistical correction of contamination must be used^8,10^.

Prior work in detecting pathogen-associated cfDNA from blood plasma has been conducted for patients in states of sepsis or other invasive or fulminant diseases^8–15^ where microorganisms directly invade the bloodstream or are capable of dissemination to that compartment secondary to disruption of endothelial and epithelial barriers, respectively. Even though detectable bacteremia is extremely uncommon in CF patients^19,20^, the extensive vascularization of the lung, the ability of some CF pathogens to express invasive functions, and the presence of inflammation-induced epithelial injury raise the possibility that cfDNA derived from lung microbes could enter the bloodstream of CF patients during chronic infection. In this exploratory, proof-of-principle study, we explored whether microbial cfDNA from common, CF-relevant pathogens is detectable in the blood plasma of CF outpatients.

## Results

### Burden of microbial cfDNA in CF patient plasma

We investigated whether plasma cfDNA from CF patients (n = 21, Table 1) showed statistical differences in the content and/or abundance of nucleic acids from selected respiratory microbes (Supplemental Table 1) relative to specimens from healthy controls (n = 12, Supplemental Table 2). Because microbial DNA is a prevalent biotechnology contaminant^8,16–18^, healthy individuals served as a control for both legitimate contamination and bioinformatic misclassification of human DNA during analysis.

**Table 1:** CF patients and clinical characteristics.

Patient ID	Age	Sex	*CFTR* genotype	Body Mass Index (BMI, kg/m²)	Forced Expiratory Volume (FEV1, L, % of predicted)	Forced Vital Capacity (FVC, L, % of predicted)	FEV1/FVC ratio
CF1	19	Female	F508del/F508del	19.52	2.01 (60%)	3.15 (83%)	0.64
CF2	39	Male	F508del/621 + G > T	24.9	3.58 (80%)	4.54 (96%)	0.66
CF3	32	Female	F508del/F508del	26.59	2.59 (80%)	3.58 (92%)	0.72
CF4	30	Female	F508del/G551D	23.12	2.72 (82%)	3.98 (102%)	0.68
CF5	28	Female	F508del/2789 + 5 G > A	18.28	1.22 (38%)	2.23 (60%)	0.55
CF6	26	Male	F508del/W1282X	20.7	2.99 (72%)	4.04 (83)	0.74
CF7	28	Male	F508del/G542X	21.74	2.13 (43%)	3.53 (60%)	0.6
CF8	55	Male	F508del/1717–1 G	21.75	0.83 (23%)	1.91 (40%)	0.43
CF9	57	Male	F508del/G551D	23.26	1.51 (42%)	3.09 (66%)	0.49
CF10	24	Female	F508del/F508del	19.68	1.84 (53%)	1.91 (47%)	0.96
CF11	29	Female	F508del/F508del	18.42	1.15 (42%)	2.0 (63%)	0.58
CF12	22	Female	W679X/unknown	22.22	1.5 (52%)	2.4 (73%)	0.63
CF13	62	Female	F508del/D1152H	23.64	1.5 (56%)	2.08 (59%)	0.72
CF14	61	Male	F508del/S945L	26.42	2.76 (37%)	3.59 (79%)	0.77
CF15	21	Female	F508del/F508del	19.44	1.26 (37%)	1.88 (48%)	0.72
CF16	35	Female	F508del/R1066C	20.07	0.92 (30%)	1.97 (53%)	0.47
CF17	33	Female	F508del/F508del	20.17	1.04 (36%)	1.73 (51%)	0.6
CF18	30	Male	F508del/R553X	17.63	1.08 (23%)	2.26 (40%)	0.48
CF19	25	Male	F508del/621 + 1 G > T	23.22	2.0 (50%)	2.67 (57%)	0.75
CF20	31	Female	F508del/R117H-5T	28.94	3.73 (111%)	4.64 (117%)	0.8
CF21	44	Female	F508del/F508del	21.9	1.96 (71%)	3.39 (100%)	0.58

We found that the abundance of microbial cfDNA from organisms of interest relative to total sequence read counts was nearly an order of magnitude higher in CF patients (1.83×10^−7^ average abundance) than in healthy controls (2.81 × 10^−8^ average abundance, p = 8.0 ×10^−3^, two-tailed t-test, Supplemental Table 3), with 14 of the 21 individual patients showing elevated microbial cfDNA at a significance of p ≤ 0.01 (two-tailed t-test).

**Figure 1:**
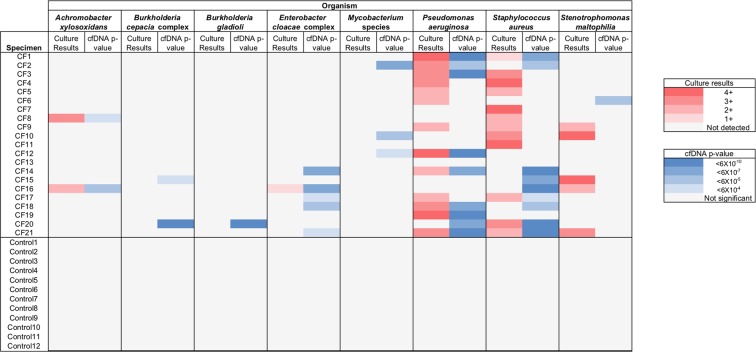
Classification of microbial sequences supported by cell-free DNA. Results of sputum microbiological culture and cfDNA testing of concordantly collected blood specimens are shown for relevant organisms. Culture results are reported according to standard 1+ to 4+ notation. cfDNA p-values were determined by Grubb’s Test for Outliers, with only values significant at p < 6×10^−4^ (the Bonferroni critical value for uncorrected p = 0.05) and supported by 2 or more reads considered valid.

In addition, whereas the plasma from 15 of 21 (71%) CF subjects contained cfDNA from one or more individual organisms at significantly greater levels than controls (one-sided Grubb’s Test for Outliers of p < 6 × 10^−4^ [the Bonferroni critical value for uncorrected p = 0.05] and supported by 2 or more reads, Fig. 1 and reported numerically in Supplemental Table 3), no healthy individual exhibited increased microbial cfDNA abundance for any of the selected organisms when tested against the remaining control set.

We found that microbial cfDNA in CF patients was more highly enriched for fragments shorter in length than the 75 bp sequencing reads (8.7%) than was human-derived cfDNA from the same specimens (1.9% of reads, p < 2.2 × 10^−16^ Pearson’s Exact test). These observations are consistent with prior reports that circulating microbial cfDNAs are more fragmented than host cfDNA^21^, and provides further support that this material originates biologically from blood plasma of CF patients.

### Comparison of microbial cfDNA to sputum microbiological culture

44 separate microbial identifications were established by clinical microbiological sputum culture (Fig. 1, reported numerically in Supplemental Table 3, and Supplemental Table 4), and 15 (34%) of these were statistically supported by cfDNA results. Plasma cfDNA was not recovered from culture-verified yeasts, molds, *Haemophilus influenzae* or *Chryseobacterium* species (11 instances, Supplemental Table 4), and was inconsistently identified from other culture-positive bacteria. Although sample sizes were small, results suggested the possibility of organism-specific variability in cfDNA recovery from culture-positive patients. 100% (2 of 2) of *Achromobacter xylosoxidans*, 100% (1 of 1) of *E. cloacae* complex, 62% (8 of 13) of *P. aeruginosa*, 33% (4 of 12) *Staphylococcus aureus*, and 0% (0 of 1) *Stenotrophomonas maltophilia* culture-positive infections were identified by sequencing. The fraction of cfDNA-positive cases that were supported by culture results were similarly varied. 100% (2 of 2) of *A. xylosoxidans*, 89% (8 of 9) *P. aeruginosa*, 44% (4 of 9) of *S. aureus*, and 20% (1 of 5) of *E. cloacae* complex cases which tested positive for cfDNA were recovered by concordant sputum culture. Because CF sputum culture may recover a mixture of both pathogenic microbes that are inflicting lung damage and also colonizing organisms that are not involved in disease^22,23^, the absence of cfDNA from microbes detected by sputum culture could be explained by organisms being present in the lungs without measurable translocation of their DNA to the circulation (low invasiveness or low pathogenicity), sputum culture recovering organisms from oropharyngeal contamination^24^ (resulting in false positives for clinically diagnosed lung infection), or false negative cfDNA results.

Conversely, 17 identifications were made exclusively by cfDNA sequencing, including *Burkholderia cepacia* complex and *Mycobacterium* species, which were not recovered by culture for any patient (Fig. 1). These results could represent true infection which was not diagnosed by sputum culture^25–27^ or false positives by cfDNA. However, analysis of healthy subjects, where microbial cfDNA is expected to be absent, did not identify control individuals exhibiting false positive results.

We further examined the 11 patient cases where classifications were only detectable by cfDNA with respect to their immediately preceding and succeeding culture results (Supplemental Table 5). For two patients (CF15 and CF18), *S. aureus* classifications identifiable only by cfDNA sequencing were recovered by succeeding culture, while for one additional patient (CF16) *S. aureus* ascertained by cfDNA was found in both the prior and succeeding microbiological cultures. The remaining 14 microbial classifications were not identified in cultures that were temporally adjacent to the time of cfDNA sequencing.

## Discussion

New methods are needed to identify the onset and persistence of CF infections, particularly those involving *P. aeruginosa*, for which existing eradication regimes improve clinical outcomes. Detection of microbial cfDNA is a novel paradigm for diagnosing infectious disease, but to date has only been used as a marker for fulminant infection^8,9^. Recovering microbial cfDNA from CF patients, where fulminant infection and overt bacteremia is absent, presents additional challenges. Here, through unbiased sequencing of cfDNA collected from blood plasma of CF patients and healthy volunteers, we explored whether cfDNA could be identified from respiratory pathogens carried in the lungs of CF outpatients.

Our findings indicate that cfDNA from relevant microbes are detectable from the circulation of CF patients at statistically greater abundance than unaffected individuals. Because many of the organisms of interest in CF are also common contaminants of low-biomass microbiome studies^28^, it was anticipated that no analyzed specimen would be entirely free of microbial cfDNA, including those from healthy donors, whom are expected to be negative for microbial cfDNA of biological origin (Fig. 1, Supplemental Table 3). Our conclusions consequently rely on comparing results from individual patients against those obtained from a population of healthy individuals who served as a control for contaminating background sequences. We found that CF patients had nearly 10-fold greater relative abundance of microbial cfDNA from CF-relevant organisms than healthy controls, and separately, multiple classifications for specific, individual organisms achieved statistical significance when tested across multiple patients. In contrast, none of the healthy control subjects showed significant levels of microbial cfDNA from any of the selected organisms, reinforcing the impact of these findings.

A subset of CF patients (33%) did not carry a significantly elevated overall relative abundance of microbial cfDNA in their plasma compared to healthy controls, and significantly elevated abundances of one or more specific organisms were not seen in a slightly smaller patient subset (29%). It is possible that these cases reflect an unusually low ratio of bacterial to human cfDNA, and that microbial cfDNA could be recovered by deeper sequencing. Alternatively, these could represent transient or constitutional biological differences among CF patients that cause cfDNA to be variably present or absent, such as acute disease state, degree of lung damage, composition of the respiratory microbiome, or patient physiology. Although our results remain exploratory, there was no significant difference between patients who were positive or negative for microbial cfDNA with respect to age, sex, BMI, FEV1, FVC, or FEV1/FVC ratio (Table 1).

Results from cfDNA showed relatively poor correlation with sputum culture, with some diagnoses made exclusively by one method or the other (Fig. 1, Supplemental Table 3 and Supplemental Table 4). However, it is known that sputum culture inconsistently identifies pathogens present in the patient airway^25–27^ and can also recover colonizing organisms that are not actively involved in disease^22–24^, making culture an imperfect comparator for evaluating either the positive or negative diagnostic accuracy of microbial cfDNA in CF patients. The short fragment length of pathogen-derived cfDNA, as found by us and others^21^, and its inconsistent coverage across microbial genomes makes it difficult to confirm its detection using orthogonal methods such as targeted PCR. It therefore cannot be known from these data whether observed discrepancies represent deficiencies of metagenomic cfDNA sequencing or culture-based diagnosis, the participation of organisms in infection or their status as colonizers^22,23^, or some combination of these factors. It is also possible that there exist organism-specific differences in the production and recoverability of microbial cfDNA.

Although encouraging, this pilot study is exploratory in nature and remains limited in several respects. First, a restricted sample size and culture positivity rate precludes robust statistical support for cfDNA as a biomarker of CF infection. Both patient and healthy donor specimens were obtained by convenience sampling, and are not in any way selected, matched, or cross-controlled. Second, we do not have access to “gold standard” diagnostic data that defines the true presence or absence of lung infections (e.g. multi-lobe bronchoalveolar lavage), making it impossible to resolve discrepancies between cfDNA and culture results or to estimate positive and negative predictive values of cfDNA testing. Third, patient material originated from routine outpatient clinic visits in the absence of pulmonary exacerbation or other emergent health issues. We therefore cannot address whether cfDNA fluctuates over time or changes with differing disease states of a given patient.

Despite its limitations, this study reveals that cfDNA from CF relevant microbes are detectable from the circulation of individual CF patients at far higher levels than observed in healthy controls. Our results provide evidence that plasma cfDNA from airway pathogens has the potential to be used in the noninvasive diagnosis of at least some chronic CF lung infections. Additional work involving larger cohorts monitored longitudinally will be needed to refine and validate methods, to establish the performance characteristics of cfDNA in identifying specific pathogens, to compare cfDNA to orthologous sputum testing methods such as 16 s rRNA profiling or metagenomic sequencing^29,30^, and to determine if the abundance of pathogen cfDNA changes during exacerbations or periods of increased lung injury.

## Methods

### Patient specimens

Specimens and clinical data were obtained according to the declaration of Helsinki and the ethics guidelines of the University of Washington Human Subjects Division. All experimental protocols were approved by the University of Washington Human Subjects Division, and informed consent was obtained from all participants. CF patients were recruited from outpatient clinics, but were otherwise not selected with respect to any clinical characteristics or inclusion criteria and consequently reflect a convenience sample (Table 1). A convenience sample of healthy volunteers without acute disease were recruited from the general population (Supplemental Table 2).

Whole blood was collected from subjects in Cell-Free DNA Blood Collection Tubes (Streck) and processed within 3 hours. Patient sputum samples collected at the time of clinic visits were subjected to microbiological culture by the University of Washington Clinical Microbiology Laboratory according to standard clinical procedures, which are described in detail elsewhere^29^.

### Library preparation and Sequencing

Plasma DNA was extracted using QIAamp Circulating Nucleic Acid Kit (Qiagen), and sequencing libraries constructed by dual-indexed Ovation Ultralow System V2 library preparation kit (NuGEN) according to manufacturer instructions. Sequencing utilized a NextSeq500 (Illumina) with 75 bp single-end chemistries and ~20 million reads allocated per sample.

### Data analysis

Sequence reads were classified using CLOMPv1.01 (https://github.com/rcs333/CLOMP) with strict tiebreaking logic. Classifications from organisms detected by microbiological culture and/or relevant to CF (n = 17, listed in Supplemental Table 1) were considered of interest, and expressed as relative abundance of total read counts per specimen.

Given the relatively small sampling size for control specimens, formal tests for normality are underpowered. However, assuming that DNA fragments are sequenced independently, at random, and that the relative abundance of background microbial reads are roughly equivalent across specimens, the relative abundance of microbial reads recovered in controls can be reasonably approximated by a normal distribution. We therefore used Grubb’s Test for Outliers, a common approach for identifying statistical outliers in data that are normally distributed, to determine whether the relative abundance of nucleic acids from select bacterial pathogens in individual CF samples were unusually high when compared to orthologous measurements from the control population. Statistical significance of individual classifications for which one or more sequence read was recovered were assessed relative to the control population using a one-sided Grubb’s Test for Outliers, with p < 6×10^−4^ (the Bonferroni critical value for uncorrected p = 0.05) and ≥2 independent supporting sequence reads required for a classification to be considered significant. Pathogen classifications were confirmed by sequence read alignment against reference genomes with manual inspection. Post-classification adapter read trimming was performed with BBDuk (https://github.com/BioInfoTools/BBMap/blob/master/sh/bbduk.sh), setting partial adapter k-mer = 6. Read length analysis used all valid pathogen classifications and 1,000 randomly selected human reads per sample.

Testing for correlation between clinical features and the presence or absence of significant microbial cfDNA levels was carried out using 2-tailed t-tests to determine whether each quantitative feature (age, BMI, FEV1, FVC, FEV1/FVC) was significantly different between groups of patients in which microbial cfDNA was detected and those in which it was not detected. Fisher’s Exact test was used to determine whether there were significant differences in the proportion of patients from different sexes between the groups. Due to the heterogeneity of genotypes represented, statistical testing was not performed for that feature.

## Supplementary information

Supplementary information.

## Data Availability

Untrimmed sequence reads supporting bacterial classifications are available from the NCBI Sequence Read Archive (SRA) under BioProject accession PRJNA578933.
