# Supplementary material for: Cell free DNA from respiratory pathogens is detectable in the blood plasma of Cystic Fibrosis patients
Source: Sci Rep. 2020 Apr 23;10:6903. doi: 10.1038/s41598-020-63970-0 (PMC7181635; doi:10.1038/s41598-020-63970-0)
Supplement: Supplementary file 1 — Supplementary information. [file 41598_2020_63970_MOESM1_ESM.pdf]

Cell free DNA from respiratory pathogens is detectable in the blood plasma of Cystic Fibrosis patients

Sara L. Rassoulian Barrett[1], Elizabeth A. Holmes[1], Dustin R. Long[2], Ryan C. Shean[1], Gilbert E. Bautista[3], Sumedha Ravishankar[3], Vikas Peddu[1], Brad T. Cookson[1,3], Pradeep K. Singh[3], Alexander L. Greninger[1], Stephen J. Salipante[1*]

Author Information:

[1]Department of Laboratory Medicine, University of Washington, Seattle, WA, USA

[2]Division of Critical Care Medicine, Department of Anesthesiology and Pain Medicine, University of Washington, Seattle, WA, USA

[3]Department of Microbiology, University of Washington School of Medicine, Seattle, WA, USA

*Address correspondence to Stephen J. Salipante, stevesal@uw.edu

Supplementary Table 1. Relevant organism classifications examined in this study

Achromobacter xylosoxidans
Aspergillus species
Burkholderia cepacia complex
Burkholderia gladioli
Candida species
Chryseobacterium species
Enterobacter cloacae
Haemophilus influenzae
Mycobacterium species
Pseudallescheria boydii
Pseudomonas aeruginosa
Scedosporium apiospermum
Scedosporium boydii
Serratia marcescens
Staphylococcus aureus
Stenotrophomonas maltophilia
Trichosporon species

Supplementary Table 2. Characteristics of control population

Sample Name	Age	Sex
Control1	53	M
Control2	35	M
Control3	50	M
Control4	21	M
Control5	35	F
Control6	21	M
Control7	35	M
Control8	36	F
Control9	38	M
Control10	46	M
Control11	38	M
Control12	54	M

Supplementary Table 3. Classificiation of microbial sequences supported by cell-free DNA

cfDNA Classifications

		Achromobacter xylosoxidans				*Burkholderia cepacia* complex				*Burkholderia gladioli*				*Enterobacter cloacae* complex			
Specimen	Total Valid Reads	Culture Results	Fraction of Reads	Count of Reads	p-value*	Culture Results	Fraction of Reads	Count of Reads	p-value	Culture Results	Fraction of Reads	Count of Reads	p-value	Culture Results	Fraction of Reads	Count of Reads	p-value
CF1	1.06E+07		0	0			0	0			0	0			0	0	
CF2	1.30E+07		0	0			7.72E-08	1			0	0			0	0	
CF3	2.62E+07		7.63E-08	2			0	0			0	0			3.82E-08	1	
CF4	2.45E+07		0	0			0	0			0	0			0	0	
CF5	7.55E+06		0	0			0	0			0	0			0	0	
CF6	2.91E+06		0	0			0	0			0	0			0	0	
CF7	6.52E+07		0	0			0	0			0	0			3.07E-08	2	
CF8	4.42E+07	3+, 3+, 3+**	**4.07E-07**	**18**	**5.29E-04**		0	0			0	0			2.26E-08	1	
CF9	2.68E+07		0	0			0	0			0	0			0	0	
CF10	1.63E+07		0	0			0	0			0	0			0	0	
CF11	1.94E+07		1.03E-07	2			0	0			0	0			0	0	
CF12	3.05E+07		3.28E-08	1			0	0			0	0			0	0	
CF13	3.29E+07		0	0			0	0			0	0			0	0	
CF14	2.39E+07		1.67E-07	4			4.18E-08	1			0	0			**2.51E-07**	**6**	**5.35E-08**
CF15	2.61E+07		1.53E-07	4			7.66E-08	**2**	**4.97E-04**		0	0			3.83E-08	1	
CF16	2.82E+07	2+	**6.04E-07**	**17**	**9.00E-06**		7.10E-08	2			0	0		1+	**2.13E-07**	**6**	**3.20E-07**
CF17	3.09E+07		3.23E-08	1			0	0			0	0			**1.29E-07**	**4**	**6.25E-05**
CF18	2.93E+07		0	0			0	0			0	0			**1.37E-07**	**4**	**3.60E-05**
CF19	3.52E+07		0	0			0	0			0	0			0	0	
CF20	3.64E+07		2.75E-08	1			6.60E-07	**24**	**5.20E-14**		5.50E-08	**2**	**<1.00E-20**		8.25E-08	3	
CF21	2.51E+07		7.97E-08	2			0	0			0	0			**1.20E-07**	**3**	**1.39E-04**
Control1	2.91E+07		6.87E-08	2			0	0			0	0			0	0	
Control2	2.08E+07		1.92E-07	4			0	0			0	0			0	0	
Control3	5.24E+07		7.63E-08	4			0	0			0	0			3.82E-08	2	
Control4	3.10E+07		6.45E-08	2			0	0			0	0			3.22E-08	1	
Control5	2.26E+07		8.85E-08	2			0	0			0	0			0	0	
Control6	6.41E+07		0	0			1.56E-08	1			0	0			1.56E-08	1	
Control7	2.73E+07		3.67E-08	1			0	0			0	0			0	0	
Control8	2.98E+07		0	0			0	0			0	0			0	0	
Control9	2.66E+07		0	0			3.75E-08	1			0	0			0	0	
Control10	1.86E+07		5.37E-08	1			0	0			0	0			0	0	
Control11	1.97E+07		0	0			0	0			0	0			0	0	
Control12	3.47E+07		0	0			0	0			0	0			2.88E-08	1	

	Mycobacterium species				*Pseudomonas aeruginosa*				*Staphylococcus aureus*				*Stenotrophomonas maltophilia*			
Specimen	Culture Results	Fraction of Reads	Count of Reads	p-value	Culture Results	Fraction of Reads	Count of Reads	p-value	Culture Results	Fraction of Reads	Count of Reads	p-value	Culture Results	Fraction of Reads	Count of Reads	p-value
CF1		0	0		4+	**8.53E-07**	**9**	**1.01E-12**	1+	**1.04E-06**	**11**	**2.88E-08**		9.47E-08	1	
CF2		1.54E-07	**2**	**9.35E-08**	3+	**2.32E-07**	**3**	**1.94E-06**		**5.40E-07**	**7**	**5.03E-05**		7.72E-08	1	
CF3		0	0		3+	**6.87E-07**	**18**	**1.12E-11**	3+	7.63E-08	2			1.15E-07	3	
CF4		0	0		3+	8.17E-08	2		4+	4.08E-08	1			8.17E-08	2	
CF5		1.32E-07	1		2+	0	0		2+	0	0			0.00E+00	0	
CF6		0	0		2+	0	0			3.44E-07	1			**6.87E-07**	**2**	**1.22E-06**
CF7		0	0			3.07E-08	2		4+	1.07E-07	7			4.60E-08	3	
CF8		0	0			0	0		2+	1.13E-07	5			1.13E-07	5	
CF9		0	0		2+	0	0		2+	1.49E-07	4		2+	1.49E-07	4	
CF10		**1.23E-07**	**2**	**1.05E-06**		0	0		3+	1.84E-07	3		3+, 4+ **	6.14E-08	1	
CF11		0	0			5.15E-08	1		4+	2.06E-07	4			1.03E-07	2	
CF12		**6.56E-08**	**2**	**5.23E-04**	4+	**5.24E-07**	**16**	**2.27E-10**		6.56E-08	2			6.56E-08	2	
CF13		0	0			3.04E-08	1			9.12E-08	3			3.04E-08	1	
CF14		0	0		2+	**3.76E-07**	**9**	**9.15E-09**		**5.73E-06**	**137**	**<2.20E-16**		4.18E-08	1	
CF15		0	0			3.83E-08	1			**1.19E-06**	**31**	**6.47E-09**	4+	3.83E-08	1	
CF16		0	0			0	0			**1.60E-06**	**45**	**2.17E-10**	2+	1.42E-07	4	
CF17		0	0		2+	0	0		2+	**4.53E-07**	**14**	**3.44E-04**		6.47E-08	2	
CF18		0	0		3+	**3.76E-07**	**11**	**9.40E-09**		**7.17E-07**	**21**	**2.08E-06**		3.41E-08	1	
CF19		0	0		4+, 3+ **	**1.73E-06**	**61**	**<2.20E-16**		3.69E-07	13			0.00E+00	0	
CF20		0	0			**3.57E-07**	**13**	**1.63E-08**	3+	**1.54E-06**	**56**	**3.32E-10**		5.50E-08	2	
CF21		0	0		3+	**1.31E-06**	**33**	**8.66E-15**	2+	**2.95E-06**	**74**	**2.11E-13**	3+	3.19E-07	8	
Control1		3.44E-08	1			0	0			1.37E-07	4			6.87E-08	2	
Control2		0	0			0	0			1.44E-07	3			9.59E-08	2	
Control3		0	0			3.82E-08	2			7.63E-08	4			9.54E-08	5	
Control4		0	0			0	0			9.67E-08	3			3.22E-08	1	
Control5		0	0			4.43E-08	1			4.43E-08	1			0.00E+00	0	
Control6		0	0			0	0			3.12E-08	2			0.00E+00	0	
Control7		0	0			3.67E-08	1			0	0			0.00E+00	0	
Control8		0	0			3.35E-08	1			1.34E-07	4			1.01E-07	3	
Control9		0	0			0	0			1.50E-07	4			1.13E-07	3	
Control10		0	0			0	0			0	0			1.07E-07	2	
Control11		0	0			0	0			1.01E-07	2			1.52E-07	3	
Control12		0	0			2.88E-08	1			2.88E-08	1			2.88E-08	1	

*By Grubb's Test for Outliers. Only values significant at p<6X10-4 (the Bonferonni critical value for uncorrected p=0.05) and supported by 2 or more reads are shown.

**Multiple colony morphologies with separate abundance measurements are reported separated by commas.

Supplementary Table 4. Organisms identified by culture that were not recovered by cfDNA analysis

Patient	Classification(s)
CF2	10 colonies *Aspergillus fumigatus* , 3+ *Candida albicans*
CF5	4+ *Haemophilus influenzae*
CF7	>30 colonies Presumptive *Trichosporon* species
CF9	3 colonies *Aspergillus fumigatus*
CF10	18 colonies *Aspergillus fumigatus*
CF14	1 colony *Aspergillus fumigatus*
CF15	>30 colonies *Aspergillus fumigatus*
CF16	2+ *Chryseobacterium* species
CF17	5 colonies *Pseudallescheria boydii* complex
CF21	2+ *Haemophilus influenzae*

Supplementary Table 5. Prior and Subsequent microbiological culture results

Patient	Days to prior culture	Prior culture results*						Days to subsequent culture	Subsequent culture results								
CF1	-42	4+ Pseudomonas aeruginosa	4+ Pseudomonas aeruginosa	4+ Pseudomonas aeruginosa	4+ Pseudomonas aeruginosa	3+ Contaminating oro-pharyngeal flora		150	4+ Pseudomonas aeruginosa	4+ Pseudomonas aeruginosa	3+ Pseudomonas aeruginosa	One colony Aspergillus fumigatus	4+ Contaminating oro-pharyngeal flora				
CF2	-154	1+ Pseudomonas aeruginosa	1+ Pseudomonas aeruginosa	15 colonies Aspergillus fumigatus	3+ Contaminating oro-pharyngeal flora			254	4+ Pseudomonas aeruginosa	2+ Pseudomonas aeruginosa	1+ Ralstonia species	3+ Contaminating oro-pharyngeal flora					
CF3	-221	3+ Pseudomonas aeruginosa	3+ Pseudomonas aeruginosa	3+ Pseudomonas aeruginosa	4+ Contaminating oro-pharyngeal flora			136	3+ Pseudomonas aeruginosa	3+ Pseudomonas aeruginosa	4+ Contaminating oro-pharyngeal flora						
CF4	-175	4+ Staphylococcus aureus	4+ Pseudomonas aeruginosa	4+ Pseudomonas aeruginosa	4+ Contaminating oro-pharyngeal flor			189	2+ Staphylococcus aureus	3+ Pseudomonas aeruginosa	3+ Haemophilus influenzae	4+ Contaminating oro-pharyngeal flora					
CF5	-91	4+ Staphylococcus aureus	4+ Pseudomonas aeruginosa	2+ Pseudomonas aeruginosa	4+ Contaminating oro-pharyngeal flor			210	4+ Staphylococcus aureus	4+ Pseudomonas aeruginosa	3+ Contaminating oro-pharyngeal flora						
CF6	-224	2+ Pseudomonas aeruginosa	2+ Pseudomonas aeruginosa	One colony Aspergillus fumigatus	4+ Contaminating oro-pharyngeal flora			175	2+ Pseudomonas aeruginosa	4+ Contaminating oro-pharyngeal flora							
CF7	-371	4+ Staphylococcus aureus	3+ Pseudomonas aeruginosa	3+ Trichosporon mycotoxinivorans	4+ Contaminating oro-pharyngeal flora			471	1+ Trichosporon mycotoxinivorans	4+ Staphylococcus aureus	4+ Contaminating oro-pharyngeal flora						
CF8	-133	3+ Pseudomonas aeruginosa	3+ Achromobacter xylosoxidans	2+ Staphylococcus aureus	4+ Contaminating oro-pharyngeal flora			266	1+ Achromobacter xylosoxidans	1+ Achromobacter xylosoxidans	1+ Elizabethkingia species	4+ Contaminating oro-pharyngeal flora					
CF9	-623	3+ Staphylococcus aureus	3+ Pseudomonas aeruginosa	4+ Contaminating oro-pharyngeal flora				21	3+ Stenotrophomonas maltophilia	6 colonies Aspergillus fumigatus	3+ Pseudomonas aeruginosa	4+ Contaminating oro-pharyngeal flora					
CF10	-175	3+ Stenotrophomonas maltophilia	15 colonies Aspergillus fumigatus	3+ Staphylococcus aureus	4+ Contaminating oro-pharyngeal flora			315	1+ Staphylococcus aureus, small colony variant	3+ Stenotrophomonas maltophilia	2 colonies Mould	3+ Contaminating oro-pharyngeal flora					
CF11	-361	4+ Staphylococcus aureus	4+ Contaminating oro-pharyngeal flora					105	1+ Staphylococcus aureus	4+ Contaminating oro-pharyngeal flora							
CF12	-144	4+ Pseudomonas aeruginosa	4+ Pseudomonas aeruginosa	4+ Contaminating oro-pharyngeal flora				196	4+ Pseudomonas aeruginosa	3+ Contaminating oro-pharyngeal flora							
CF13	-126	4+ Staphylococcus aureus	4+ Staphylococcus aureus	3+ Pseudomonas aeruginosa	4+ Contaminating oro-pharyngeal flora			217	4+ Staphylococcus aureus	1+ Pseudomonas aeruginosa	4+ Contaminating oro-pharyngeal flora						
CF14	-133	4+ Pseudomonas aeruginosa	3+ Pseudomonas aeruginosa	3+ Pseudomonas aeruginosa	3+ Pseudomonas aeruginosa	3 colonies Aspergillus fumigatus	4+ Contaminating oro-pharyngeal flora	189	1+ Pseudomonas aeruginosa	3 colonies Aspergillus fumigatus	4+ Contaminating oro-pharyngeal flora						
CF15	-741	4+ Stenotrophomonas maltophilia	11 colonies Aspergillus fumigatus	4+ Contaminating oro-pharyngeal flora				217	3+ Staphylococcus aureus	3+ Stenotrophomonas maltophilia	>30 colonies Aspergillus fumigatus	4+ Contaminating oro-pharyngeal flora					
CF16	-214	1+ Staphylococcus aureus	3+ Achromobacter xylosoxidans	3+ Achromobacter xylosoxidans	One colony Aspergillus fumigatus	2+ Contaminating oro-pharyngeal flora		196	One colony Enterobacter asburiae	2+ Stenotrophomonas maltophilia	3+ Staphylococcus aureus	2+ Chryseobacterium species	3+ Staphylococcus aureus	3+ Staphylococcus aureus	2+ Acinetobacter bereziniae	3+ Achromobacter xylosoxidans	3+ Contaminating oro-pharyngeal flora
CF17	-144	4+ Staphylococcus aureus, small colony variant	>10 colonies Pseudallescheria boydii complex	3+ Contaminating oro-pharyngeal flora				206	1+ Staphylococcus aureus	>30 colonies Scedosporium apiospermum complex	4+ Contaminating oro-pharyngeal flora						
CF18	-280	3+ Pseudomonas aeruginosa	3+ Pseudomonas aeruginosa	4+ Contaminating oro-pharyngeal flora				276	4+ Pseudomonas aeruginosa	3+ Staphylococcus aureus	2+ Stenotrophomonas maltophilia	1+ Pseudomonas aeruginosa	3+ Contaminating oro-pharyngeal flora				
CF19	-190	1+ Escherichia coli	4+ Pseudomonas aeruginosa	4+ Pseudomonas aeruginosa	4+ Pseudomonas aeruginosa	3+ Contaminating oro-pharyngeal flora		160	4+ Pseudomonas aeruginosa	4+ Pseudomonas aeruginosa	3+ Contaminating oro-pharyngeal flora						
CF20	-140	4+ Staphylococcus aureus	1+ Pseudomonas species	4+ Contaminating oro-pharyngeal flora				322	4+ Contaminating oro-pharyngeal flora	4+ Staphylococcus aureus							
CF21	-133	1+ Staphylococcus aureus	2+ Pseudomonas aeruginosa	2+ Pseudomonas aeruginosa	2+ Pseudomonas aeruginosa	4+ Contaminating oro-pharyngeal flora		253	3+ Stenotrophomonas maltophilia	3+ Stenotrophomonas maltophilia	3+ Pseudomonas aeruginosa	3+ Pseudomonas aeruginosa	4+ Contaminating oro-pharyngeal flora				

*Multiple colony morphologies with separate abundance measurements are reported separately.
